# Effect of driving characteristics and ambient temperature on the particle emissions during engine restart of spark ignition hybrid electric vehicle

**DOI:** 10.1038/s41598-023-44497-6

**Published:** 2023-10-13

**Authors:** Yonghyun Choi, Joonsik Hwang, Sungwook Park

**Affiliations:** 1 Department of Mechanical Engineering, Mississippi State University, Mississippi State, MS 39762 USA; 2 Center for Advanced Vehicular Systems (CAVS), Starkville, MS 39759 USA; 3 School of Mechanical Engineering, Hanyang University, 222 Wangsimni-ro, Seongdong-gu, Seoul, 04763 Republic of Korea

**Keywords:** Mechanical engineering, Pollution remediation

## Abstract

In this study, we analyzed particle emission characteristics in the engine restart (ER) phase of a hybrid electric vehicle (HEV) based on driving characteristics and ambient temperature. The ambient temperature was set at intervals of 10 °C from − 10 °C to 20 °C. ES-582.1, PPS-M, EEPS, and temperature sensors were installed to acquire hybrid control unit (HCU), particle number (PN), PN size distribution, and exhaust temperature data. The on board test route was conducted in the South Korean real driving emissions (RDE) certification route, consisting of urban, rural, and motorway phases. The test HEV was controlled by dividing the engine operation during driving into ER and normal phases. Within 5 s immediately after ER, it emitted PN equivalent to 90% of the total test emissions. The count of ER was higher in urban phases compared to rural and motorway phases. As the ambient temperature decreased, PN emissions increased regardless of the driving mode, but the ER PN percent decreased. Immediately after ER, PN emissions increased rapidly, peaked at around 2–3 s, and then decreased thereafter. The average engine-off time before ER was the longest in the urban phase, and the average ER exhaust temperature was the highest in the motorway phase. The size fraction of large particles increased as the ambient temperature decreased.

## Introduction

Climate change events such as global warming are accelerating due to greenhouse gases such as CO_2_ emitted from vehicles^1–3^. In addition, harmful emissions from vehicles pollute the atmosphere and have a detrimental effect on human health^4–9^. For that reason, the need for carbon neutrality has recently increased, and many studies on high efficiency and high fuel efficiency are in progress to reduce CO_2_ and harmful emissions. To reduce CO_2_, gasoline direct injection (GDI) engines account for a significant portion of gasoline vehicles. Because GDI engines have the advantage of increasing fuel economy and torque compared to PFI engines^10–14^.

The international energy agency (IEA) organized the net zero emission (NZE) policies for automobiles around the world and made a scenario. As a result, it was mentioned that most of the raw materials for electric vehicle batteries are in short supply, and particular, lithium, a major raw material, is short of about 40% of the required amount, making the NZE policy impossible^15^. At the same time, the IEA recommended the development of carbon capture utilization (CCU) technology or coexistence with internal combustion engines using E-fuel and said that hybrid electric vehicle (HEV)s were essential. Since HEV can achieve high fuel efficiency compared to conventional internal engine combustion vehicles, their share in the current market is gradually increasing^16–20^.

HEVs consist of a combination of an engine and a motor, and power is distributed through the hybrid control unit (HCU). Because the motor assists the power, HEVs consume less fuel compared to the conventional internal combustion engine (ICE)^21–23^. In some low-load conditions, the car is driven only by the motor, and the engine operates as the load increases, so the engine turns on and off continuously while driving^24^. For this reason, engine restart (ER) events frequently occur in HEVs. When the engine is restarted, a lot of gaseous and particle emissions are emitted in this section. Reference^25^ repeatedly performed real driving emissions (RDE) experiments using PFI HEVs. As a result, it was found that the emission of CO and PN was remarkable when the engine was restarted. Reference^26^ conducted an RDE test on a total of four vehicles, conventional engine vehicles using PFI and GDI engines and HEV using PFI and GDI engines. As a result, much more PN was emitted from hybrid vehicles than conventional engine vehicles in both PFI and GDI vehicles, and they did not satisfy the RDE regulations in China and Europe. Although it was found that a lot of particles are emitted during restart, there is no research related to the fundamental consideration of the causal relationship according to the most important combustion strategy in engine combustion.

Important factors that have a great influence on hazardous emission is ambient temperature. Reference^27^ found that the total PN emission was about 49% higher in winter than in summer as a result of experimenting with plug-in hybrid electric vehicle (PHEV) under summer (24–29 °C) and winter (10–14 °C) conditions. Reference^28^ analyzed start-stop, a phenomenon similar to HEV’s engine restart, through a worldwide harmonized light-duty vehicle test procedure (WLTP) chassis dynamometer experiment for a vehicle equipped with a GDI engine. As a result, it was found that when the engine is restarted in the start-stop on state, more particles are emitted at an ambient temperature of 5 °C than at a condition of 28 °C. There are several studies conducted about ambient temperature and emissions for gasoline vehicles other than HEV. Reference^6, 9^ analyzed the gaseous and particle emission characteristics emitted when using gasoline and ethanol-blended gasoline in WLTP at ambient temperatures of − 7 and 30 °C with GDI vehicles. They found that all measured hazardous emissions except fuel consumption and NO_x_ increased significantly when the ambient temperature was lowered from 30 °C to − 7 °C, and particulate emissions from all fuels increased significantly with decreasing temperature. The IUFC15 cycle was tested at outdoor temperatures of 23 °C, − 7 °C, and − 20 °C, and CO and HC emissions during cold start increased significantly at lower ambient temperatures^29^. Although ambient temperature has a great effect on particle emission, there has been no study related to engine restart of HEV. In addition to the ambient temperature, there is a driving characteristic as a factor that has a great influence on the emission. Reference^30^ analyzed the emission characteristics according to the actual road test for not-plug-in hybrid vehicles and NG vehicles. As a result, it was analyzed that NG vehicles always had lower PN emissions than HEV. And the effect of ICE restart on the PN emission trace was highlighted. However, a clear analysis of the effect of driving path characteristics (urban, rural, motorway) was not performed.

A lot of studies related to hybrids have been conducted mainly related to fuel economy and energy-saving^31–34^. However, there is a lack of studies on the influence of ambient temperature and driving characteristics related to the engine restart section, which is a fatal disadvantage of HEV. Therefore, in this study, particle emission characteristics as a function of ambient temperature and driving characteristics were analyzed of the GDI HEV. The on board test was carried out by loading the measuring equipment inside the vehicle and driving it on the actual road. PN emissions were analyzed in the normal section and the restart section in which the outside air temperature was changed in 10 °C increments from − 10 °C to 20 °C.

## Experimental apparatus and conditions

### Test vehicle

The 1600 cc model was sold the most among light-duty vehicles (LDV), and the best-selling HEV was selected as the test vehicle. The intake of the test vehicle is equipped with a turbocharger, and a side mount type GDI engine is installed. It is a mild HEV vehicle that regenerates while driving or charges by running the engine without the need to charge the battery separately. The maximum power of the engine is 134 kW, and the maximum power of the motor is 44 kW. The gasoline particulate filter (GPF), a filter that reduces particulate emissions, was not attached to the exhaust of the test vehicle. Detailed information on the test vehicle is shown in Table 1. All experiments were conducted with one GDI HEV.

**Table 1:** Specification of the test vehicle.

Items	Specifications
Powertrain type	Hybrid
Engine fuel-supply system	GDI
Intake system	Turbo-charged
Displacement (cc)	1598
Engine maximum power (kW)	134
Motor maximum power (kW)	44
Battery type	LiPB
Model year	2021
Weight (kg)	1590
Transmission	6-speed automatic transmission

### On board particle measurement test

The on board test route in this study was conducted in the South Korean RDE certification route that satisfies the RDE-LDV standard^35, 36^. The experiment route reflects various characteristics of general roads and consists of three consecutive routes, urban, rural, and motorway. The share for each section is Urban 34%, Rural 33%, and Motorway 33%. The urban section minimum share must be over 29%, and the minimum mileage per section must be 16 km. There is a speed limit for each section, Urban is 60 km/h or less, Rural is 60 ~ 90 km/h and Motorway is 90 ~ 110 km/h. The on board test was conducted for 90 to 120 min. The test route is the same as the (b) Route B in Fig. A of the^35^.

**Figure 1:**
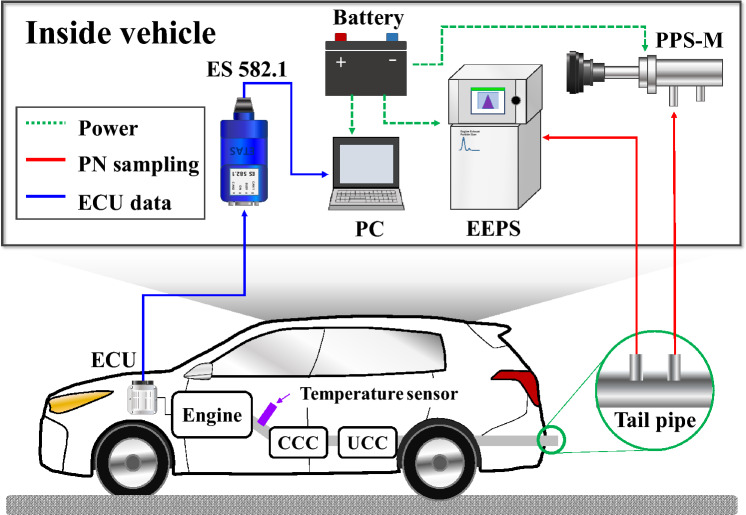
Schematic diagram of the on board test particle measurement system.

The schematic diagram of the on board test in this study is shown in Fig. 1. In this study, a Pegasor particle sensor (PPS-M, Pegasor, Finland) and an engine exhaust particle sizer (EEPS-3090, TSI, USA) were used to measure PN by sampling some of the exhaust gas from the tailpipe. The two instruments have a slightly different measurement range for particle size. In the case of the PPS-M, it can measure the number of particles between 23 nm and 2.5 μm, and in the case of the EEPS, it can measure the number of particles between 5.6 nm and 560 nm. Both instruments use a similar corona discharge method to measure particles, and the PPS measures particles collectively in one channel. However, the EEPS is divided into 32 channels, and by measuring particles of different sizes for each channel, not only the number of particles but also the distribution of each particle size can be measured. The basic exhaust system of the test vehicle used in this study consisted of close-coupled catalytic converter (CCC) and under-floor catalytic converter (UCC) catalysts. The temperature sensor was installed between the engine exhaust and the CCC. The ES-582.1 (ETAS, Germany) was used to acquire the controller area network (CAN) data of the hybrid control unit (HCU) such as vehicle speed, engine speed, engine torque, motor torque, lambda, start of injection (SOI), fuel consumption and coolant temperature. The power supply of the experimental equipment was not supplied by the internal battery of the HEV, but an external battery was additionally loaded on the trunk side to supply power.

The purpose of this study is to analyze the effect of particle emission according to driving conditions and ambient temperature in on board tests. To this end, the experiment was conducted from November to April, and a total of 4 cases were conducted with an interval of 10 °C from − 10 °C to 20 °C ambient temperature. The error of the ambient temperature was ± 2 °C. In the case of the battery state of charge (SOC), the test was conducted by charging it to 65% in the same way before the start of the test. In this study, a hot start test in which the engine was preheated was conducted. In the case of the hot start test, the engine was preheated by operating the engine until the coolant temperature of the test vehicle reached 90 °C before starting the experiment. All experiments in this study were conducted under the same initial conditions.

## Results and discussion

### HEV power flow

**Figure 2:**
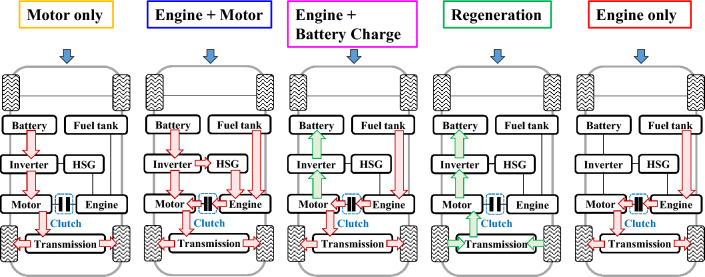
HEV power flow diagram while driving.

Figure 2 shows the power flow diagram of the research vehicle during driving. This test vehicle is a parallel-type transmission mounted electric device (TMED) mild HEV and is largely composed of a fuel tank, an engine, a hybrid starter generator (HSG), a battery, an inverter, a motor/generator, and a transmission. The HCU properly distributes power to the engine and motor and controls regenerative braking based on the driver’s request, vehicle condition, engine information, and battery information. When driving a vehicle, it is largely controlled by the HCU in five modes depending on the driving characteristics: motor only, engine + motor, engine + battery charge, regeneration, and engine only.

When the vehicle is stopped and then starts driving, or at low speed and low load, it drives only with the motor. Power is supplied from the battery to the motor through an inverter, and power is transmitted to the tires through the transmission. Not only at the beginning of driving but also in some constant speed sections or low-load acceleration sections, it is driven only by the motor. The vehicle runs only with the motor, but when the load increases, such as in acceleration or high-speed sections, the engine turns on and runs with the engine and motor. At this time, the HSG, which plays a role similar to the starting motor of a conventional internal combustion engine vehicle, rotates the engine and raises it until it becomes the same as the driving rpm of the motor, connects to the clutch, and then turns off. And when the combustion of the engine starts, power is transmitted along with the motor. HEV, unlike BEV, periodically recharge their batteries to maintain them in a stable condition. When the SOC of the battery is low during driving or at a standstill, it is replenished through engine operation. During this period, the electric motor does not provide propulsion power to the vehicle; instead, it functions as a generator. During deceleration through brake application while driving, a regenerative braking process is engaged to enhance the SOC of the battery. During driving, only the engine is utilized in the section where the engine efficiency is good. It is shown in detail in the power flow diagram through the arrow in Fig. 2.

### HEV engine restart combustion strategy

**Figure 3:**
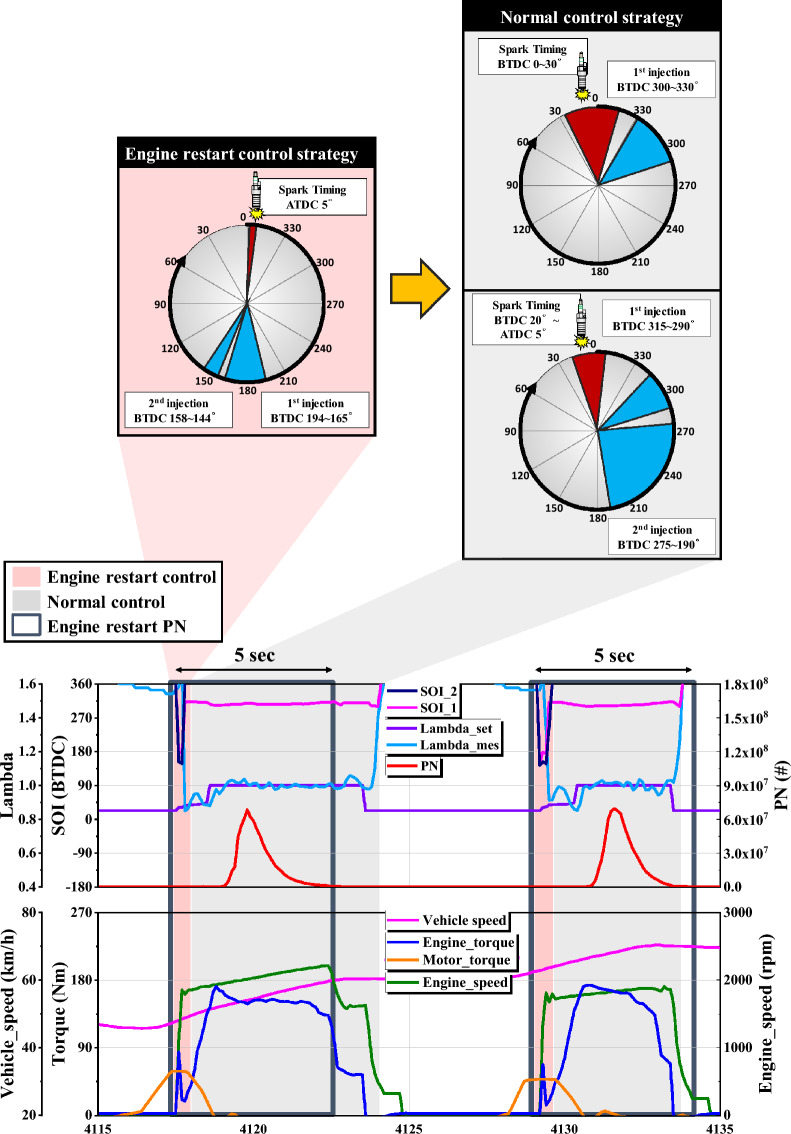
GDI HEV driving control strategy.

**Figure 4:**
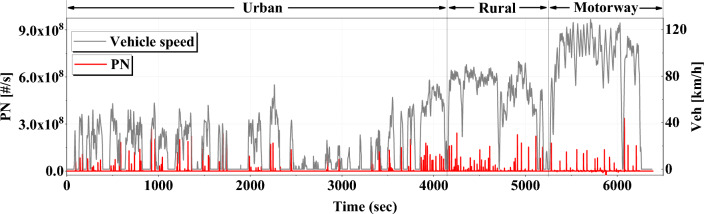
Vehicle speed and PN emission profiles of on-board test.

Previous studies have confirmed that HEVs emit many harmful substances immediately after ER^25, 26^. Therefore, in this study, the analysis was conducted focusing on the ER section combustion strategy. Figures 3 and 4 were analyzed based on data acquired during on-board test of HEV under the condition of an ambient temperature of 20 °C. The upper part of Fig. 3 shows the injection and spark timing for each control section of GDI HEV. The lower part of Fig. 3 is an enlarged graph of some sections of the on board test and shows the engine control strategy and PN emissions. This graph shows lambda, start of injection, PN, vehicle speed, engine torque, motor torque, and engine speed data.

When the engine is running, the test HEV divides the combustion control strategy into two sections, the ER control section, and the normal control section. The ER control section is controlled for a short period of time immediately after the ER, and then, until right before the engine turns off, it is controlled as a normal control section. Zones shaded in red represent ER control zones and gray shading represents normal engine control zones.

When the engine is restarted, it is similar to a cold start. This is because combustion does not occur from the time the engine is turned off until it is restarted, so the combustion chamber is cooled. Therefore, it is difficult to atomize and vaporize the fuel in the initial stage of restarting the engine. And since combustion does not occur and then suddenly and rapidly occurs, a stable combustion strategy is required. During the compression stroke, the volume decreases and the internal temperature rises. Therefore, when fuel is injected at this time, atomization and vaporization of the fuel are promoted, and it is advantageous to form a fuel-rich mixture around the spark plug, so that stable combustion can be expected. However, if the injection timing is delayed too much, the air–fuel mixture formation time is too short, rather increasing the possibility of incomplete combustion, and there is a problem in that many particles are emitted, so fuel is injected at the beginning of the compression stroke^37, 38^. When the engine is restarted, it uses a strategy of injecting fuel at the beginning of the compression stroke for a short time of about 0.2 s. In the ER section control, fuel is double injected between BTDC 194 ~ 165 deg and BTDC 158 ~ 144 deg, and the ignition timing is at ATDC 5 deg. Unlike the ER section control, the normal section control injects fuel at the beginning of the intake stroke. In the beginning, when the load is low, a single injection is performed, but at high loads where a lot of fuel is injected, a double injection is performed as shown in the Fig. 3.

When fuel is injected immediately after restarting the engine, it is injected under control at lambda 0.88, which is a slightly fuel-rich condition than the theoretical air–fuel ratio. This is because combustion occurs stably under slightly rich-fuel conditions. As fuel is injected during the compression stroke, mixture formation time is short and wall wetting increases. Due to the rich fuel injection strategy, a large amount of PN is emitted within 5 s immediately after ER. In this study, we named this ER PN and marked it with a navy box. Through the vehicle speed data and motor torque data, it can be seen that the test HEV drives only with the motor at the beginning of acceleration, then restarts the engine as the load increases. When the engine is restarted, HEV’s HSG rotates the engine, causing the engine torque to temporarily rise and fall rapidly. Then, as the amount of combustion inside the engine increases, the engine torque increases again. Figure 4 is a graph showing the driving speed and PN emission overtime during the on-board experiment. As described above, it can be confirmed that PN is rapidly emitted a lot when the engine is restarted.

### HEV on board test PN by section

A significant amount of PN was emitted during the 5 s immediately after ER, and to analyze how much PN was emitted during driving, the analysis was divided into sections as shown in Table 2. The section was divided into an ER PN and a normal PN. The PN data used here was analyzed based on the PPS data. ER count is the total count of engine restarts during the on board test, ER PN time was calculated by multiplying the count of restarts by 5 s, and ER PN is the sum of all PN emitted within 5 s from the time of ER. Of the total time, the ER PN time was 11.3% and the normal PN time was 88.7%, which was relatively much longer in the normal PN section. The ER PN was 90.2%, and the normal PN was 9.8%. Calculating PN emissions per hour was 72.54 times higher in the ER PN section than in the normal PN section. Therefore, it can be seen that a fairly large amount of PN is emitted per hour in the ER section.

**Table 2:** GDI HEV on board test section analysis.

Items	ER PN	Normal PN	Total
ER count	152	–	–
Time [sec]	760 (11.3%)	5283 (88.7%)	6721 (100%)
PN [#]	1.02 × 10^11^ (90.2%)	1.1 × 10^10^ (9.8%)	1.13 × 10^11^ (100%)
PN [%]/time [%]	7.98	0.11	1

### HEV on board test PN by ambient temperature

**Figure 5:**
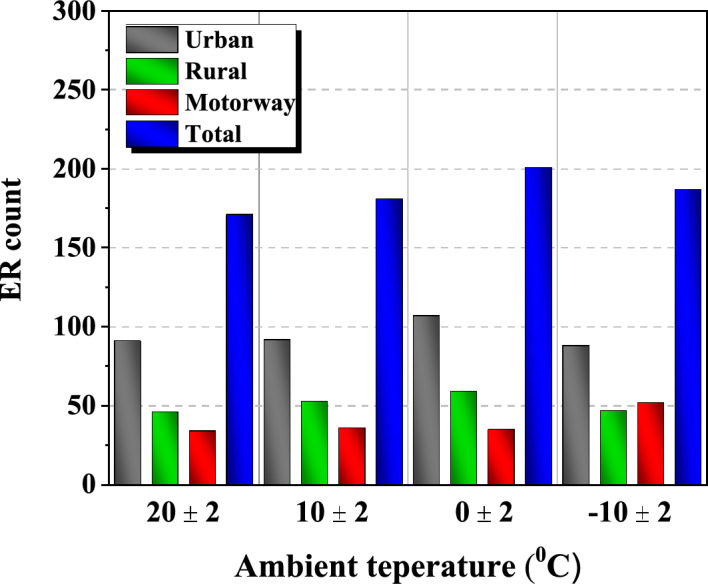
ER count according to the ambient temperature.

**Figure 6:**
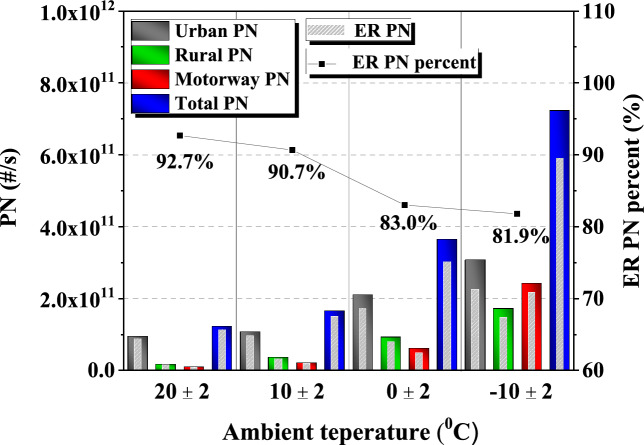
PN by phase, ER PN, ER PN percentage according to the ambient temperature.

The on board test is divided into urban, rural, and motorway phases and consists of various driving patterns. In this study, the PN emission characteristics according to these various driving patterns were analyzed. Figures 5 and 6 are experimental data under hot start conditions where the engine is preheated to 90 °C before the test. A total of 4 cases were conducted by changing the ambient temperature at 10 °C intervals between − 10 °C and 20 °C.

Figure 5 shows the count of ER in each phase and the total. When the outside ambient temperature was 20, 10, 0, − 10 °C, the urban section ER count ratio to the total ER count was 53.2%, 50.8%, 53.2%, and 47.1%, accounting for about half. The count of ER was similar in the rural and motorway phases or slightly higher in the rural phases. Due to the nature of the urban phase, there are many traffic lights and vehicles, so brakes are often used and the speed limit is low. HEV mainly turns off the engine when decelerating or stopping and activates when accelerating^26^. Like this, in an urban phase where brakes are used a lot and low-speed and low-load driving conditions are the most common, vehicle ER events occur very frequently^39^. On the other hand, rural and motorway phases have relatively few traffic lights and high-speed limits, resulting in a high engine-drive ratio and continuous operation, resulting in relatively fewer ER compared to the urban phase.

Figure 6 shows the percentage of total PN emissions and ER PN emissions by phase. 'Urban PN, rural PN, motorway PN’ refers to the total amount of PN emitted for each phase during on board test for each ambient temperature case, and 'ER PN' refers to the sum of PN emissions for 5 s immediately after ER. To compare how much ER PN occupies PN emission in each phase, ER PN is superimposed on the PN emission graph bar. This was also analyzed based on the PPS data. When comparing PN emissions by ambient temperature in Fig. 6, phase, total PN emissions increase as ambient temperature decreases. This is because, as the ambient temperature decreases, liquid atomization and mixture formation decreases, which prevents complete combustion. The percentage occupied by ER PN tends to decrease from 92.7 to 81.9% as the ambient temperature changes from 20 °C to − 10 °C. From this, it can be seen that the lower the ambient temperature, the higher the PN emission rate in the section where the engine is running, excluding the restart section. Comparing the trend of the graphs in Figs. 5 and 6, the graph of the count of ER by phase and the graph of PN emissions by phase show similar trends except for the ambient temperature conditions. For all temperatures, PN emissions are particularly high in the urban phase because ER events are more frequent. This shows that the count of ER has a significant effect on PN emissions.

However, simply comparing PN emissions to ambient temperature and driving characteristics may not be accurate. This is because the greater the count of ER events, the greater the PN emission. ${ER \,PN}_{av}$ was defined in this paper to perform an accurate analysis by excluding the effect of PN emissions according to the count of ER.

1 $${ER \,PN}_{av}= ER \,PN/ER \,\,number.$$

**Figure 7:**
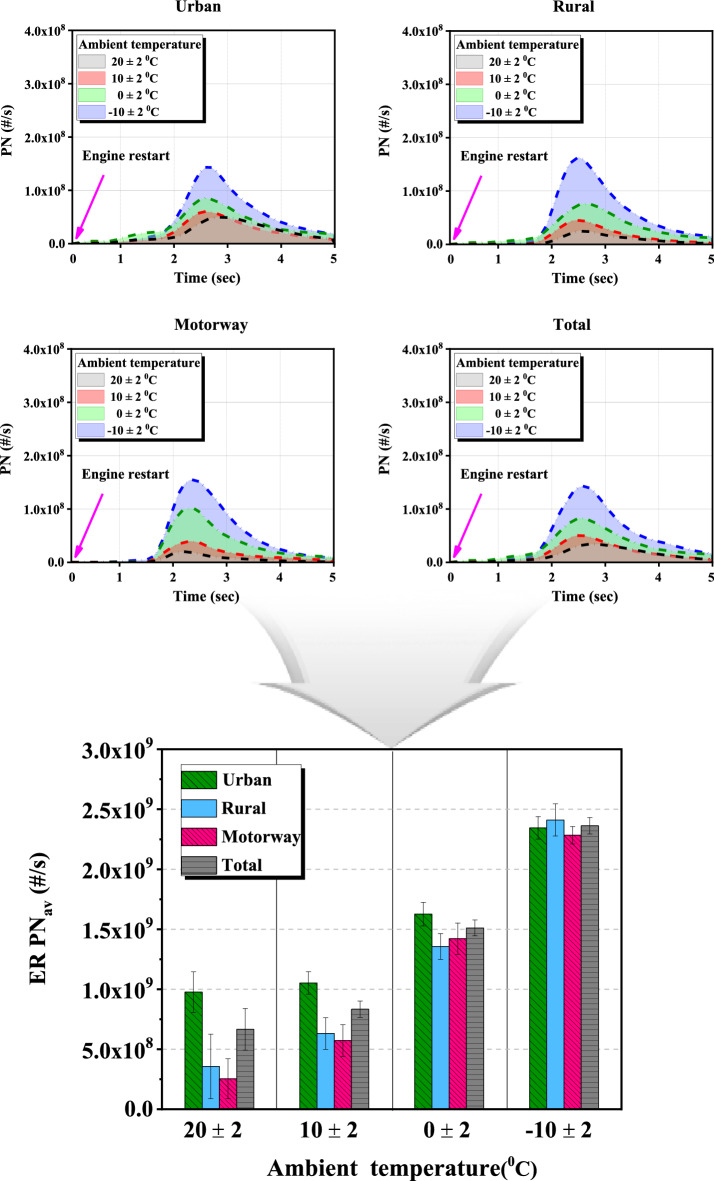
Phase and total PN emission average over time, ${ER \,PN}_{av}$ according to the ambient temperature.

**Figure 8:**
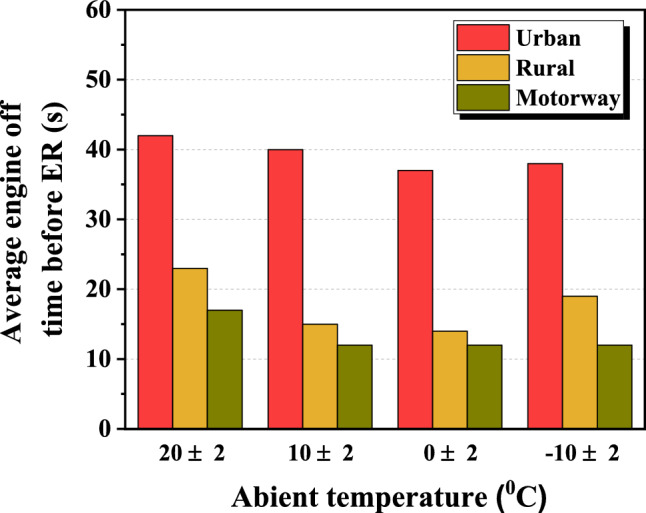
Average engine-off time before ER according to the ambient temperature.

**Figure 9:**
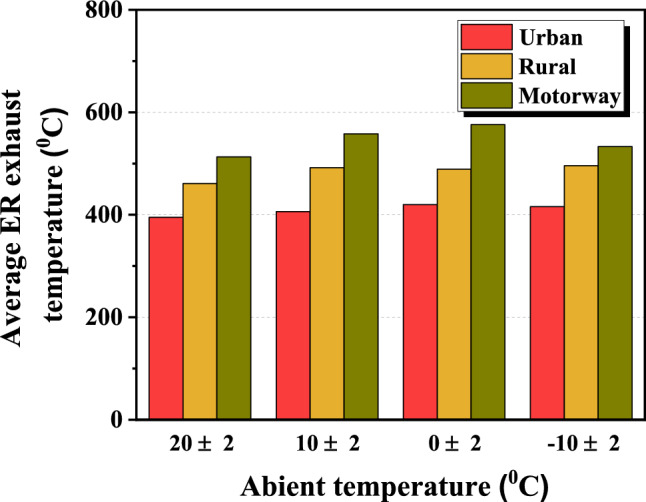
Average ER exhaust temperature according to the ambient temperature.

Through INCA, it can be acquired through a variable indicating whether the engine restart mode is in operation in the HCU. Based on this data, the ER timing was identified, and through Matlab code the PN data emitted for 5 s immediately after ER was extracted, summed and divided by the count of ER. By calculating as ${ER \,PN}_{av}$ in Eq. (1), it is possible to compare the effect of PN emission according to ambient temperature or driving condition regardless of the count of ER in the HEV. The upper four graphs in Fig. 7 show the time-resolved PN average emissions immediately after ER according to the ambient temperature based on the PPS data in the hot start ER section, and the lower graph shows the ${ER \,PN}_{av}$ value as a bar graph. Figures 8 and 9 show the average engine-off time before ER and the average ER exhaust temperature which are measured between the engine and the CCC immediately after the ER.

Looking at the time-resolved PN average emission of Fig. 7, it can be seen that it increases rapidly immediately after ER, shows the highest emission between 2 and 3 s, and then decreases thereafter. In addition, the PN emission area increased as the ambient temperature decreased regardless of the phase. As shown in Fig. 8, the average engine-off time before ER of each phase was 39.3 s, 17.7 s, and 13.3 s, respectively, which is about twice as long in the urban phase compared to the other phases. The reason is that traffic lights and low-speed driving cause cars to stop longer and the engine to shut off longer when comparing the urban phase to the rural and highway phases. In addition, the motorway section has the least amount of time for the engine to be turned off. The engine is used more frequently because the engine is more efficient at this section. As shown in Fig. 9, the average ER exhaust temperatures of each phase were 409.3 °C, 484.5 °C, and 545 °C, respectively, indicating that the engine in the urban phase was relatively cool compared to other phases. This is because, as described above, the engine does not operate relatively longer due to low-speed driving of 60 km/h or less in the urban section and frequent stops at traffic lights.

When the ambient temperature is 20 °C, the ${ER \,PN}_{av}$ value is higher in the urban section compared to other sections. As the ambient temperature dropped from 20 °C to − 10 °C, the average ${ER \,PN}_{av}$ ratios of the rural and motorway sections to the ${ER \,PN}_{av}$ of the urban section gradually increased to 31.3%, 57.2%, 85.4%, and 100.1%. As the ambient temperature is lower, the intake air temperature is lowered to prevent the evaporation of fuel or the formation of a mixture, and since a large amount of PN is discharged even in a section other than a restart section, the influence on driving characteristics is reduced.

### HEV on board test PN size distribution by ambient temperature

**Figure 10:**
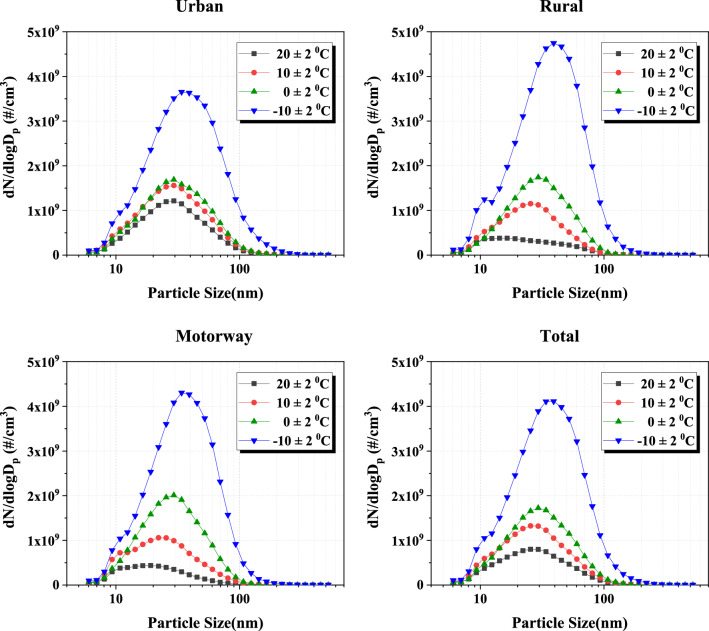
PN size distribution according to ambient temperature for each driving mode.

**Figure 11:**
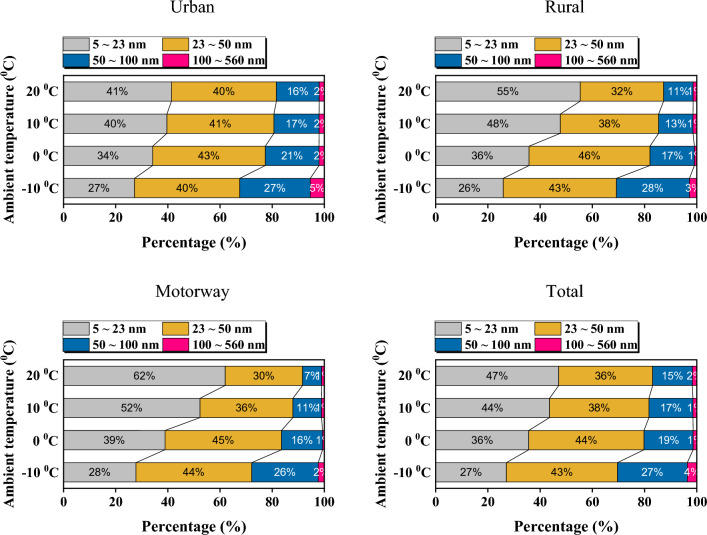
Classified by particulate diameter according to ambient temperature for each driving mode.

Figure 10 shows the PN size distribution as a function of ambient temperature based on the EEPS data of the ER segment for each driving mode. From the total size distribution graph, it can be observed that as the ambient temperature decreases, there is a general increase in the emission of particles. At low-temperature conditions such as − 10 °C, the combustion becomes challenging due to the lower ambient temperature, leading to a tendency for a significant increase in particle size. Under conditions of 20 °C and 10 °C in urban section, there is a relatively higher emission of PN compared to rural and motorway sections. This is because, in urban section, combustion is less active compared to other sections due to driving characteristics. On the other hand, the rural and motorway phases have relatively less engine stop time and the amount of fuel injection is high due to the high load compared to the urban phase. These driving characteristics result in significant engine preheating and ensure smooth combustion. Figure 11 shows the classified by particulate diameter according to the ambient temperature based on the EEPS data in the hot start ER section. As the ambient temperature decreases from 20 °C to − 10 °C, the total fraction of accumulation mode particles between 50 and 1000 nm increases from 17 to 31%. In general, as the ambient temperature decreases, the proportion of large particles increases^40^, and the proportion of accumulation mode particles between 50 and 1000 nm, which occur mainly in fuel-rich regions, also increases^41, 42^. This is because when the ambient temperature is low, the fuel does not evaporate well and a smooth mixture is not formed, increasing the local fuel-rich area^43^. At 20 °C condition, the ratio of accumulation mode particles differs from 18% to urban, 13% to rural, and 8% to motorway. This is considered to be different because the engine is warmed up more on the motorway.

In the urban stage of the hot start, the proportion of PNs with a size of over 23 nm, which is the current emission regulation particle size, accounted for about 60% at an ambient temperature of 20 °C, and increased by about 14% as the ambient temperature decreased from 20 °C to − 10 °C. On the other hand, in the rural phase and the motorway phase of hot start, the proportions of PNs with a size of over 23 nm accounted for about 45% and 38%, respectively, when the ambient temperature was 20 °C. As the ambient temperature decreased from 20 °C to − 10 °C, the number of particles larger than 23 nm increased rapidly by approximately 30%.

## Conclusion

In this study, the effect of the driving condition and ambient temperature on particle emission characteristics were analyzed through on board testing of a GDI HEV. Finally, the conclusions we have drawn are as follows.

1. Throughout the on-board testing, the test HEV was segregated into an ER control section and a normal control section. Upon analyzing PN emissions, it was determined that approximately 90% of the total emissions occurred within the 5 s following ER.
2. On-board testing revealed that ER events occurred more frequently during the urban phase compared to the rural and motorway phases, irrespective of ambient temperature. As ambient temperature decreased, total PN emission increased, and the ER PN percent decreased from 92.7 to 81.9%.
3. As the ambient temperature decreased, there was a tendency for the variation in ${ER \,PN}_{av}$ based on driving conditions to decrease.
4. The average engine-off time before ER was the longest in the urban phase, and the average ER exhaust temperature was the highest in the motorway phase. Regardless of the driving condition, the ratio of large particles tended to increase as the ambient temperature decreased.

HEV technology are widely recognized as a significant solution for greenhouse gas reduction and environmentally friendly transportation by various research institutions and nations worldwide. Regarding particle emissions from HEV, additional research is expected to be needed on the impact of GPF attachment and restart control strategies, which were not covered in this study. In addition, it is expected that various research topics will be actively explored, such as design aspects, power distribution, and control strategies to improve fuel efficiency. Through this, HEV technology is expected to continue to develop and become an important component of future eco-friendly automobile technology.

## Abbreviations

ATDC: After top dead center
BEV: Battery electric vehicle
BTDC: Before top dead center
CAN: Controller area network
CCC: Close-couple catalytic converter
CCU: Carbon capture utilization
CO: Carbon monoxide
CO_2_: Carbon dioxide
EEPS: Engine exhaust particle sizer
ER: Engine restart
GDI: Gasoline direct injection
GPF: Gasoline particulate filter
HC: Hydrocarbons
HCU: Hybrid control unit
HEV: Hybrid electric vehicle
HSG: Hybrid starter generator
ICE: Internal combustion engine
IEA: International energy agency
LDV: Light-duty vehicles
LNT: Lean NO_x_ trap
NZE: Net zero emission
NO_x_: Nitrogen oxides
PFI: Port fuel injection
PN: Particulate number
PPS: Pegasor particle sensor
RDE: Real driving emissions
SCR: Selective catalytic reduction
SOC: State of charge
SOI: Start of injection
TMED: Transmission mounted electric device
WLTP: Worldwide harmonized light-duty vehicle test procedure
